# Immunometabolism of ferroptosis in the tumor microenvironment

**DOI:** 10.3389/fonc.2024.1441338

**Published:** 2024-08-12

**Authors:** Gian Luca Lupica-Tondo, Emily N. Arner, Denis A. Mogilenko, Kelsey Voss

**Affiliations:** ^1^ Department of Pathology, Microbiology and Immunology, Vanderbilt University Medical Center, Nashville, TN, United States; ^2^ Department of Medicine, Department of Pathology, Microbiology and Immunology, Vanderbilt Center for Immunobiology, Vanderbilt University Medical Center, Nashville, TN, United States; ^3^ Department of Pharmacology, University of Virginia, Charlottesville, VA, United States

**Keywords:** ferroptosis, immunometabolism, TME, iron, metastasis

## Abstract

Ferroptosis is an iron-dependent form of cell death that results from excess lipid peroxidation in cellular membranes. Within the last decade, physiological and pathological roles for ferroptosis have been uncovered in autoimmune diseases, inflammatory conditions, infection, and cancer biology. Excitingly, cancer cell metabolism may be targeted to induce death by ferroptosis in cancers that are resistant to other forms of cell death. Ferroptosis sensitivity is regulated by oxidative stress, lipid metabolism, and iron metabolism, which are all influenced by the tumor microenvironment (TME). Whereas some cancer cell types have been shown to adapt to these stressors, it is not clear how immune cells regulate their sensitivities to ferroptosis. In this review, we discuss the mechanisms of ferroptosis sensitivity in different immune cell subsets, how ferroptosis influences which immune cells infiltrate the TME, and how these interactions can determine epithelial-to-mesenchymal transition (EMT) and metastasis. While much focus has been placed on inducing ferroptosis in cancer cells, these are important considerations for how ferroptosis-modulating strategies impact anti-tumor immunity. From this perspective, we also discuss some promising immunotherapies in the field of ferroptosis and the challenges associated with targeting ferroptosis in specific immune cell populations.

## 1 Introduction

First described in 2012, ferroptosis is now an established cell death modality distinct from apoptosis, pyroptosis, and other regulated cell death pathways (1). The discoveries which led to the classification of ferroptosis as a distinct and unique form of cell death are described in detail elsewhere (2). Briefly, ferroptosis is an iron-dependent, caspase-independent process characterized by an accumulation of lipid-based reactive oxygen species (ROS) (3). Iron reacts with oxygen and membrane lipids containing polyunsaturated fatty acids (PUFAs) to generate lipid peroxides. These lipid peroxides accumulate in excess, and plasma membrane rupture and death occur due to weakened membrane integrity. Although necroptosis and pyroptosis also result in plasma membrane rupture, ferroptosis rupture is not due to pore-forming proteins and instead relies on specific lipid biology. Ultimately, the increase in membrane lipid peroxide species is required for the execution of ferroptosis and is considered the key defining feature of ferroptosis.

The cell signaling and regulation of ferroptosis involves multiple pathways that ultimately shape the ferroptosis sensitivity of a given cell (4). Central to all ferroptosis is the generation of membrane-bound PUFAs oxidized by free iron-mediated Fenton chemistry or iron-containing enzymes. Therefore, lipid peroxidation can occur non-enzymatically or enzymatically. In Fenton reactions, free labile (II) iron reacts with peroxides to create hydroxyl radicals and ferric (III) iron. In enzymatic lipid peroxidation, iron-containing enzymes such as lipoxygenases (LOX) mediate peroxidation and their activation status depends on the cellular redox state (5). Therefore, iron is central to driving ferroptotic lipid peroxidation either directly or indirectly. In addition to iron metabolism, however, multiple pathways integrate to regulate ferroptosis including lipid metabolism, ROS and glutathione peroxidase 4 (GPX4), the system x_c_^−^, and the mevalonate pathway (6, 7).

Understanding how to trigger various forms of cells death in the setting of cancer is of high importance and interest for anti-cancer therapy. Thus, the discovery of ferroptosis spurred a wave of excitement with the potential to both cause cancer cell death in cancers that are resistant to other forms of treatment, and potentially promote inflammation in the tumor microenvironment (TME) to enhance anti-tumor immunity. After further study, roles for ferroptosis in cancer progression can be described as serving both pro- and anti-tumorigenic properties. The precarious role of ferroptosis in cancer is likely due to the complex regulation of iron and lipid metabolism in the TME, affecting cancer and immune cells differently (8). For example, while suppressing tumor growth in a variety of blood and solid tumors, the induction of ferroptosis through GPX4 knockout or iron-feeding can accelerate KRAS-mutant pancreatic ductal adenocarcinoma (9). Thus, the immunometabolism of the immune cells within the TME should also be considered when envisioning ferroptosis therapies in the clinic. Here, we discuss the complex interplay between the metabolic states of cells in the TME, what is known about their potential metabolic vulnerabilities to ferroptosis, and how this may ultimately influence cancer progression and metastasis.

### 1.1 Iron metabolism

Intracellular iron accumulation is essential for the progression of ferroptosis. One of the tests of cell death by bona fide ferroptosis is that treatment of the cells with iron chelators suppress cell death. However, due to the potentially damaging redox potential of iron, its levels in cells are subject to complex and tight regulation (10). In most cells, iron enters as ferric (Fe^+3^) iron bound to transferrin (Tf) protein. Tf-bound iron is endocytosed after binding to the transferrin receptor (TfR1), also referred to as CD71 on immune cells (11). Iron is reduced in the endosome upon acidification to ferrous (Fe^+2^) iron by the reductase STEAP3. The ferrous iron can then be pumped into the cytosol by the divalent metal transporter 1 (DMT-1). Most ferrous iron is stored within ferritin protein in the cytosol unless it is labile iron.

In cases of iron starvation and/or increased demand for iron in the cell, a selective autophagy process called ferritinophagy occurs to release cellular iron stores from ferritin protein. The nuclear receptor coactivator 4 (NCOA4) protein acts as an autophagic receptor that targets ferritin for degradation. This process releases ferrous iron that can maintain mitochondrial respiration (12). Cellular iron levels are also tightly regulated in feedback loops by iron regulatory proteins (IRP)s. IRPs function by binding to iron-responsive elements (IREs) in the untranslated regions of target messenger RNAs (mRNAs), affecting their translation or stability (13). Depending on iron availability in the cell, iron binds to RNA-binding proteins (RBP) creating a positive or negative feedback loop on transcripts for TfR1, ferritin subunits, or other iron handling proteins. Therefore, when iron levels are sufficient, IRP binding to TfR1 transcripts decreases, resulting in its degradation and decreased receptor production (14). RBP also regulate systemic iron homeostasis. IRP1 is particularly crucial for erythropoiesis and iron absorption by regulating hypoxia-inducible factor 2α (HIF2α) translation, whereas IRP2 primarily controls iron uptake and heme biosynthesis in erythroid cells by modulating the expression of TfR1 and 5-aminolevulinic acid synthase 2 (ALAS2) mRNAs (15).

Iron levels are controlled at the tissue and systemic level by hormones, immune cells, and metabolism. Central to this regulation is the Hepcidin-Ferroportin axis, which acts to prevent the systemic accumulation of iron (16). Ferroportin is the only known exporter of iron on cells. Hepcidin produced primarily by liver hepatocytes is released into circulation and causes Ferroportin degradation. Ultimately, this lowers systemic iron as cells retain their iron stores. This axis plays an important role in the innate immune response by sequestering iron from pathogens (17). For example, activated macrophages release IL-6, triggering hepcidin production in the liver and resulting in iron sequestration from pathogenic bacteria. Interestingly, hepcidin levels increase in some non-bacterial infections such as in SARS-CoV-2 (COVID-19) patients, suggesting potentially conserved inflammatory responses that modulate Hepcidin (18).

Macrophages in the spleen and liver regulate iron recycling. Specifically, red pulp macrophages and Kupffer cells phagocytose senescent or damaged erythrocytes, metabolize heme and recycle iron (19). Furthermore, tissue-resident macrophages in adipose tissue, skeletal muscle, and skin have been implicated in local iron handling, acting as ‘ferrostats’ that modulate iron levels in specific tissue niches (20). Overall, immune cells are instrumental in regulating iron availability in tissues throughout the body at baseline and during infectious challenge, and are subject to tight regulation for their own intracellular iron stores.

### 1.2 Lipid metabolism

Metabolic enzymes like ELOVL fatty acid ligase 5 (ELOVL5), and fatty acid desaturase 1 and 2 (FADS1 and FADS2) mediate the synthesis of unsaturated PUFAs such as linoleic acid and α-linolenic acid. PUFAs are subjected to processes of elongation and desaturation to produce longer, more unsaturated fatty acids that are prone to peroxidation, thus increasing substrates for ferroptosis. Ether lipids, including PUFA-containing ether phospholipids, are drivers of ferroptosis (21). Conversely, monounsaturated fatty acids (MUFAs), like palmitoleic acid and oleic acid, which are synthesized by fatty acid synthase (FASN) from malonyl-CoA and acetyl-CoA, are less susceptible to peroxidation. Therefore, increased MUFAs in cell membranes can displace the more oxidizable PUFAs, reducing lipid peroxidation potential and ferroptosis sensitivity. Enhancing MUFA synthesis by overexpression of stearoyl-CoA desaturase 1 (SCD1), activation of mammalian target of rapamycin complex 1 (mTORC1), or inhibition of AMP-activated protein kinase (AMPK), can increase resistance against ferroptosis (22). Therefore, this dynamic between PUFA and MUFA metabolism plays a critical role in determining a cell’s vulnerability to ferroptosis (23).

The long-chain acyl-CoA synthetase (ACSL) enzyme family, including ACSL4, is essential in incorporating PUFAs into cell membranes. ACSL4 activates long-chain PUFAs by esterifying coenzyme A (CoA) followed by their incorporation into membrane-bound phospholipids, which can then undergo peroxidation via labile iron, resulting in ferroptosis (24). Mechanistically, lipid peroxidation of ACSL4-activated PUFAs occurs through two major pathways: hydrogen atom transfer (HAT) and peroxyl radical addition (PRA). Briefly, HAT involves the peroxyl radical accepting a hydrogen atom from a donor molecule, while PRA involves the peroxyl radical adding itself to a double bond (25).

Although PUFAs provide more substrates for peroxidation, not all PUFAs equally contribute to ferroptosis as some are more susceptible than others to HAT and/or PRA. Conjugated linoleic acid (CLA 18:2), for example, significantly enhanced the toxicity induced by (1S,3*R*)-Ras Selective Lethal 3 (RSL3) in the HT-1080 cell line more than other nonconjugated PUFAs, while demonstrating low toxicity on its own. The greater efficacy of CLA 18:2 in promoting ferroptosis compared to nonconjugated PUFAs is partly due to its autoxidation process, which involves both HAT and PRA, with the latter not occurring in nonconjugated PUFAs (26). Additionally, recent work compared PUFAs containing a single fatty acyl tail (PL-PUFA_1_s) or two diacyl tails (PL-PUFA_2_s) and discovered that the PL-PUFA_2_s phosphatidylcholine (PC)-PUFA_2_s generated mitochondrial ROS that initiated lipid peroxidation (27). The activity of enzymes regulating intracellular PUFA and MUFA levels directly influences the susceptibility of specific lipids to ferroptosis. This susceptibility is crucial in understanding how nutrient composition of the tumor microenvironment (TME) affects the uptake of these fatty acids by different immune cells, potentially impacting immune responses and tumor progression.

### 1.3 The system X_c_−

Glutathione (GSH) is a thiol present in all eukaryotes that is essential for oxidative metabolism. GSH is synthesized from cysteine, which is imported into the cell in its oxidized form, cystine (Cys) (28). The system x_c_− is a chloride-dependent, sodium-independent antiporter mainly consisting of a light chain, xCT, and a heavy chain (4F2hc) (29). The system x_c_− exchanges Cys to uptake and glutamate (Glu) to secrete from the cell in a 1:1 manner. After import, γ-glutamate–cysteine ligase (GCL) combines Cys and Glu to create γ-glutamylcysteine which then combines with glycine by glutathione synthetase (GSS) to produce GSH. Although GSH is generated in the cytosol, GSH can be found among many organelles such as the nucleus and the mitochondria. Compounds such as erastin that inhibit system x_c_− can lead to cysteine deprivation and increased ferroptosis sensitivity, making system x_c_− a promising target in drug-resistant tumors (30). The dependence of the system x_c_− on intracellular glutamate and extracellular cystine suggests that extracellular amino acid availability and intracellular metabolic pathways could influence the efficiency of the system, thereby modulating ferroptosis sensitivity.

### 1.4 Glutathione peroxidase 4

Glutathione peroxidase 4 (GPX4) is a key orchestrator bridging iron metabolism, lipid peroxidation, and ferroptosis. GPX4 protects cells from oxidative stress by reducing lipid hydroperoxides, thereby preventing lipid peroxidation and maintaining cellular integrity. GPX4 has been reviewed elsewhere in detail, given its central role in the ferroptosis (31, 32). Briefly, the catalytic site of GPX4 contains a cysteine that mediates oxidation-reduction reactions using GSH. First, the selenocysteine group of GPX4 becomes oxidized to selenic acid. At the same time, the lipid peroxide is reduced to a lipid alcohol. Second, the oxidized GPX4 is reduced with two equivalents of GSH, allowing for the next oxidation-reduction cycle to proceed. The net result of the reaction reduces lipid hydroperoxides into lipid alcohols.

GPX4 exists as cytosolic, mitochondrial, and nuclear isoforms. Although mitochondrial GPX4 has the most associations with inhibiting ferroptosis in cancer cells, cytosolic GPX4 is expressed in all cell types and was the only isoform able to rescue embryonic lethality of GPX4 deletion in mice (33). The increased association of mitochondrial GPX4 with cancer cells is likely due to the complex relationship between mitochondrial activity and ferroptosis (34). Mitochondrial respiration via electron transport chain (ETC) activity generates ROS through complexes I and III, promoting ferroptosis. However, GSH can also be transported into mitochondria via the SLC25A39 transporter to combat ferroptosis (35). Interestingly, SLC25A39 expression is subject to regulation by iron availability and GSH levels (36). SLC25A39 is constantly degraded by the mitochondrial protease AFG3L2, unless GSH is depleted and AFG3L2 dissociates from SLC25A39. This allows for stable expression of SLC25A39 and, thus, GSH import into the mitochondria. Furthermore, SLC25A39 also senses mitochondrial iron levels by an iron-sulfur cluster that protects it from degradation (37). Overall, the system x_c_−/GSH/GPX4 pathway connects ferroptosis, lipid metabolism, and iron metabolism and suggests a potential target for ferroptosis-inducing cancer therapies.

### 1.5 Mevalonate pathway and CoQ_10_

Other enzymes independent of GPX4 such as Coenzyme Q10 (CoQ10) can reduce lipid peroxides to suppress ferroptosis. Reduced CoQ10 traps and reduces lipid radicals relying on NADPH instead of GSH. CoQ10 is regenerated by ferroptosis suppressor protein (FSP1), which becomes anchored to the plasma membrane via myristoylation (38). Interestingly, this pathway is upregulated in various ferroptosis-resistant cancer cell lines similar to GPX4 upregulation (39, 40). Besides FSP1, selenium can also reduce mitochondrial CoQ10 via a Hydrogen Selenide intermediate and further contribute to ferroptosis resistance (41).

The mevalonate pathway synthesizes sterol isoprenoids such as cholesterol and also mediates ferroptosis by affecting both GPX4 and CoQ10 biology. First, it influences the synthesis of GPX4 by regulating the maturation of selenocysteine tRNA through adenosine isopentenylation (42). Second, this pathway is essential to produce CoQ10 by facilitating the conjugation of isoprene units to a hydroxybenzoic acid backbone. Thus, inhibiting the mevalonate pathway can reduce the synthesis of both GPX4 and CoQ10, promoting ferroptosis (43). However, many lipid products of the mevalonate pathway are also key building blocks for post-translational modifications such as N-glycosylation and protein prenylation (44).

### 1.6 Emerging pathways that contribute to ferroptosis

Cancer cell lines across different tissue types exhibit a wide range of susceptibilities to GPX4 inhibitors such as ML210, suggesting alternative pathways that regulate ferroptosis sensitivity (45). These novel regulators of ferroptosis are being increasingly discovered in a large part due to CRISPR screening technologies. To find genes that contribute to resistance, genome-wide or pooled small guide RNAs (sgRNAs) of a gene group of choice can be applied to a cell line, treated with GPX4 inhibitors, and screened for gene deletions that confer survival. Using this approach, sgRNAs targeting *FSP1* were significantly depleted in U-2 OS cells, revealing a role for FSP1 in ferroptosis (40). In another CRISPR knockout screen, pleckstrin homology-like domain family A member 2 (PHLDA2) knockout was shown to confer resistance to ferroptosis under Cys starvation (46). Furthermore, ACSL4/GPX4/PHLDA2 triple-knockout cancer cell lines were resistant to ferroptosis, indicating that the mechanism is independent of GPX4. PHLDA2 knockout alone was not lethal, suggesting that PHLDA2 metabolically sensitizes cells to ferroptosis indirectly by promoting ALOX12-mediated peroxidation of phosphatidic acid (PA). PHLDA2 recruited ALOX12 to stimulate membrane peroxidation. Therefore, this pathway is believed to induce ferroptosis in specific metabolic conditions such as high ROS or cystine deprivation.

Gene discovery can also be accomplished by CRISPR activation screens, where enhanced expression of individual genes confers survival to the cells under pressure. This type of screening approach was applied to fibrosarcoma cells under conditions of Cys starvation or GPX4 inhibition to promote ferroptosis. In this screen, known ferroptosis genes such as FSP1 and SLC7A11, encoding the xCT light chain, were strong hits for both Cys starvation and/or GPX4 inhibition (47). Interestingly, the weaker sgRNA hits, although still significant, were composed of previously unknown ferroptosis regulators such as membrane bound O-acyltransferase domain containing 2 (MBOAT2). Both MBOAT2 and MBOAT1 were shown to inhibit ferroptosis independent of other ferroptosis-promoting pathways by selectively incorporating MUFAs into the plasma membrane and simultaneously decreasing PE-PUFA abundance. Given that PUFAs are the primary substrate for ferroptosis, upregulation of these enzymes could confer resistance to several cancers (47).

## 2 Immune cells have distinct ferroptosis sensitivities

**Figure 1:**
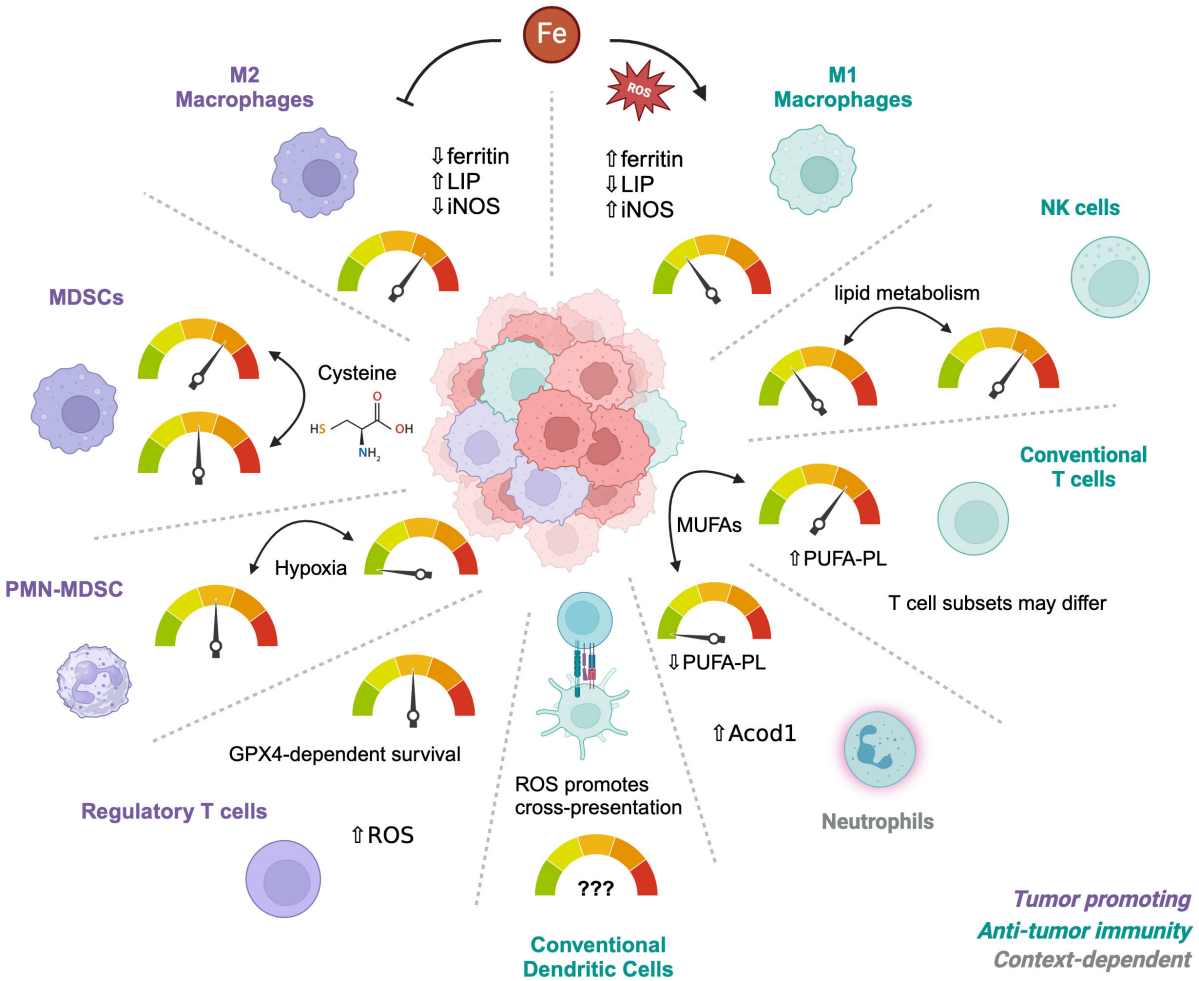
Differential sensitivities of immune cells to ferroptosis. The tumor microenvironment contains multiple types of immune cells depending on the circumstances and tumor type. Different subsets or subtypes of these cells have established differences in their sensitivity to ferroptosis, such as M1 macrophages which are more resistant than M2 macrophages. Alterations in the metabolic environment such as hypoxia, Cysteine availability, or iron concentrations can influence their relative sensitivities, depending on individual vulnerabilities. Iron metabolism is the main driver of M1 versus M2 sensitivities whereas Cysteine regulates survival of MDSCs. PMN-MDSCs become resistant under hypoxic conditions. Regulatory T cells depend on GPX4 for survival but only after activation and have adapted to a metabolism that is high in ROS at baseline. Neutrophils are naturally more resistant than T cells due to low PUFA-PL contents and these phenotypes can be reversed by MUFAs. However, the resistance of different CD4 T cell subsets is unknown.

In addition to metabolically active cancer cells, the TME is occupied with various immune cell populations with distinct metabolic demands for their survival, proliferation, and/or effector function (48, 49). Despite the interest in targeting ferroptosis for cancer therapy, how ferroptosis is regulated in immune cells remains insufficiently understood. Indeed, multiple studies now show that ferroptosis occurs in both innate and adaptive immune cells (50). One strategy to address this gap in knowledge is to perform detailed lipidomic profiling of immune cells to predict their susceptibilities to ferroptosis (51). Using this approach, a low PUFA-PL content in neutrophils successfully predicted ferroptosis resistance. By contrast, lymphoid cells were enriched in PUFA-PLs and were more sensitive to the GPX4 inhibitors compared to monocytes or neutrophils. Altering PUFA-PL content by supplementing immune cells with MUFAs such as oleate was sufficient to reverse these phenotypes, suggesting that manipulating fatty acid composition in immune cells could be a therapeutic opportunity to alter their ferroptosis sensitivities. Overall, an understanding of ferroptosis sensitivity in immune cell populations is needed to appreciate how targeting ferroptosis could influence anti-tumor immunity, metastasis and the TME (**Figure 1**).

### 2.1 Macrophages

Out of all the immune cells, perhaps the most progress in understanding iron metabolism and ferroptosis is seen in the tumor-associated macrophages (TAMs). Macrophages are important regulators of systemic iron homeostasis and are local tissue ferrostats. In the TME, however, TAMs typically provide a source of iron to cancer cells and their abundance is associated with poor prognosis (52). Macrophages exist on a spectrum of phenotypes, including classical pro-inflammatory (M1) macrophages which are anti-tumorigenic, and alternatively activated tissue-repairing (M2) macrophages which are pro-tumorigenic. However, macrophage function and phenotypes are heterogeneous and plastic, especially under the environmental pressures of the TME (53).

Iron levels can alter M1/M2 polarization, with increased iron levels promoting M1 polarization through increased reactive oxygen species (ROS) and p53 acetylation (54). Interestingly, M1 and M2 macrophages also have distinct iron handling metabolism. M1 macrophages retain or sequester iron in ferritin protein with a small labile iron pool (LIP), whereas M2 macrophages internalize, process and release iron at higher rates and have a larger LIP (55).

Given the differential iron metabolism between M1 and M2 macrophages, it is not surprising that different macrophage subsets also exhibit differential ferroptosis sensitivities (17). Generally, M1 macrophages are more resistant to ferroptosis than M2 macrophages. This could be due in part to high inducible nitric oxide synthase (iNOS) expression in M1 macrophages despite similar levels of GPX4 and ACSL4 between M1 and M2 subsets (56). iNOS produces NO^•^, and the free radical can interact with lipid peroxides and effectively quench the incorporation of lipid peroxides into the membrane. Indeed, inducing iNOS expression in M2 macrophages and inhibiting iNOS expression in M1 macrophages reversed respective sensitivities to ferroptosis (56).

Genetic models of GPX4-deficiency in macrophage (CD11c+ and LysM+) lineages have also revealed susceptibilities of macrophage subsets depending on inflammatory or non-inflammatory conditions. Macrophages populations such as monocyte-derived macrophages and tissue-resident macrophages did not rely on Gpx4 expression for development (57). Surprisingly, GPX4-deficient M1 macrophages respond similarly to TLR stimulation *ex vivo* and do not rely on Gpx4 expression for survival or cytokine production. However, GPX4-deficient M2 macrophages generated from the bone marrow experienced ferroptosis. Gpx4 was not required for M2 differentiation *in vivo* but was needed for survival during a murine hookworm infection. Interestingly, supplementation of M2 knockout cells with NO protected cells from death, but inhibition of iNOS in M1 cells was not sufficient to induce ferroptosis. Therefore, other metabolic pathways in macrophages likely converge to determine ferroptosis sensitivity in M1 versus M2 and others (57).

In glioblastoma, bone marrow derived macrophages (BMDMs) are the major immune cell population in the TME that demonstrates high metabolic activity for iron-recycling (58). Interfering with this metabolism by blocking the catabolism of heme via heme oxygenase-1 (HO-1) inhibition reduced the immunosuppressive activity of BMDMs from patients with glioblastoma (59). Treatment of patient BMDMs with the inhibitor zinc PPIX (ZnPPIX), a HO-1 inhibitor, was able to rescue T cell proliferation, which was associated with the downregulation of PD-L1 on the macrophages and reduced IL-10 secretion. In a mouse model of thymoma cells that promotes immunosuppressive TAMs, deletion of HO-1 in myeloid cells also caused PD-L1 downregulation, which occurred through epigenetic reprogramming (60).

In breast cancer, TAMs are also associated with poor prognosis and engage in metabolic cross talk with cancer cells (61). By secreting ferritin, TAMs promote cancer cell growth and metastasis (62). In a mouse model of breast carcinoma, HO-1 inhibition through ZnPPIX treatment reduced cancer growth, but also promoted a switch from M2 to M1 phenotypes in the TAMs (63). Interestingly, HO-1 activity in TAMs has also been implicated in metastasis (64). More recent work in triple-negative breast cancer (TNBC) shows how TAMs create an immunosuppressive TME by promoting ferroptosis resistance in cancer cells. In this example of cross-talk, an oncoprotein called hepatic leukemia factor (HLF) conferred ferroptosis resistance by activating gamma-glutamyltransferase 1 (GGT1) (65). HLF was directly bound to the *GCT1* promoter region in chromatin immunoprecipitation (chIP) assays. This HLF expression was dependent on TAM-derived TGF-β1 secretion, which was further supported by tumor cell IL-6 secretion. Interestingly, HLF was also predicted to have a binding site in the IL-6 promotor region suggesting a forward feedback loop of IL-6 secretion, TAM secretion of TGF-β1, and ferroptosis resistance in TNBC cells.

### 2.2 Myeloid-derived suppressor cells

Myeloid-derived suppressor cells (MDSCs) are a cell type defined by their immunosuppressive function on T cells and natural killer (NK) cells. They can be subdivided into granulocyte-like MDSCs (G-MDSCs) or mononuclear phagocyte-like MDSCs (M-MDSCs), although their defining markers and phenotypes are increasingly heterogenous and MDSCs have also been proposed as a “cell state” rather than a particular cell identity type (66). Like TAMs, an accumulation of MDSCs in tumors is usually associated with a poor prognosis, but crosstalk between TAMs and MDSCs in the TME additionally enhances both of their suppressive functions (67, 68). IL-6 is associated with MDSC accumulation and targeting IL-6 is one strategy to reduce MDSC suppression of anti-tumor immunity (69). However, the role of IL-6 signaling in the TME with respect to ferroptosis is complicated. In some cases, IL-6 from myeloid cells can promote ferroptosis resistance in cancer cells by upregulating transcription of xCT through JAK2/STAT3 signaling (70). However, depending on the cell type, IL-6 can also induce ferroptosis by disrupting iron homeostasis (71).

In colon carcinoma, an inhibitor of ceramidase (N-acylsphingosine amidohydrolase [ASAH2]) called NC06 induced ferroptosis in MDSCs and was rescued by ferroptosis inhibitors (72). ASAH2 protected MDSCs from ferroptosis by suppressing p53 signaling, which then regulated HO-1 expression. Whether MDSCs have a similar iron recycling phenotype to M2 macrophages, however, is unclear. Interestingly, MDSC accumulation in the kidney is also associated with lupus nephritis in the autoimmune disease systemic lupus erythematosus (SLE). In a murine model of SLE, induction of HO-1 signaling by a flavonoid compound baicalein reduced inflammation and prevented the accumulation of MDSCs in multiple organs compared to the healthy controls (73). Therefore, regulation of oxidative stress in MDSCs during inflammatory conditions, whether it be the TME or a systemic autoimmune disease, probably relies on HO-1 activity.

Pathologically activated neutrophil (PMN) PMN-MDSCs have been shown to undergo ferroptosis in the TME. PMN-MDSCs isolated from murine tumors were more susceptible to RSL3-induced death compared to cells isolated from spleen or bone marrow, suggesting the signals or metabolic environment in the TME promote ferroptosis sensitivity in MDSCs (74). Indeed, hypoxic conditions in the TME recapitulated the downregulation of GPX4 seen in PMN-MDSCs from the tumor compared to the spleen or bone marrow. Ferroptosis of MDSCs released oxidized phospholipids that suppressed T cell proliferation. Whether those released lipids also trigger ferroptosis in T cells is unclear. However, MDSCs may mediate inhibitory effects indirectly on T cells through other ferroptosis-promoting mechanisms. Naïve T cells lack a functional system x_c_^−^ rendering them unable to import cystine. Cystine and subsequent cysteine is required for their function and generation of GSH therefore, they must acquire it from the other cells such as DCs or macrophages during activation (75). Because MDSCs can import cystine but lack the machinery to export cysteine, they sequester the available cysteine in the extracellular environment from T cells (76). Therefore, MDSCs in the TME may be rendering T cells uniquely sensitive to ferroptosis by indirectly depleting their GSH production potential.

### 2.3 T cells

T cells and CD4 T helper (Th) cell subsets have distinct metabolic programs that are linked to their differentiation and effector function. Despite this, little is known about their iron metabolism and ferroptosis sensitivity across different subsets. In all tests of iron deprivation or CD71 blockade to date, iron uptake during T cell activation has proven important for full activation and proliferation in CD8 T cells (77), CD4 T cells (78), and mucosal-associated invariant T (MAIT) cells (79). We and others have shown that Th1 cells are most sensitive to iron withdrawal or low iron conditions with a loss of viability (80, 81), whereas Th17 cells rely on iron for differentiation and IL-17 production (82). T follicular helper (Tfh) cells also rely on iron for differentiation, which is dependent on epigenetic regulation in a mouse model of lupus (83). Indeed, in multiple models of autoimmunity, restraining iron ameliorated CD4 T cell-mediated pathology through metabolic rewiring, epigenetic mechanisms, or altered mitochondrial function (80, 82, 84). On the other hand, iron overload in patients with myelodysplastic syndrome (MDS) displays lower percentages of T cells, which contain high levels of ROS (85). This dichotomy highlights the critical balance of iron levels in regulating T cell function, differentiation, and, ultimately, adaptive immunity.

**Figure 2:**
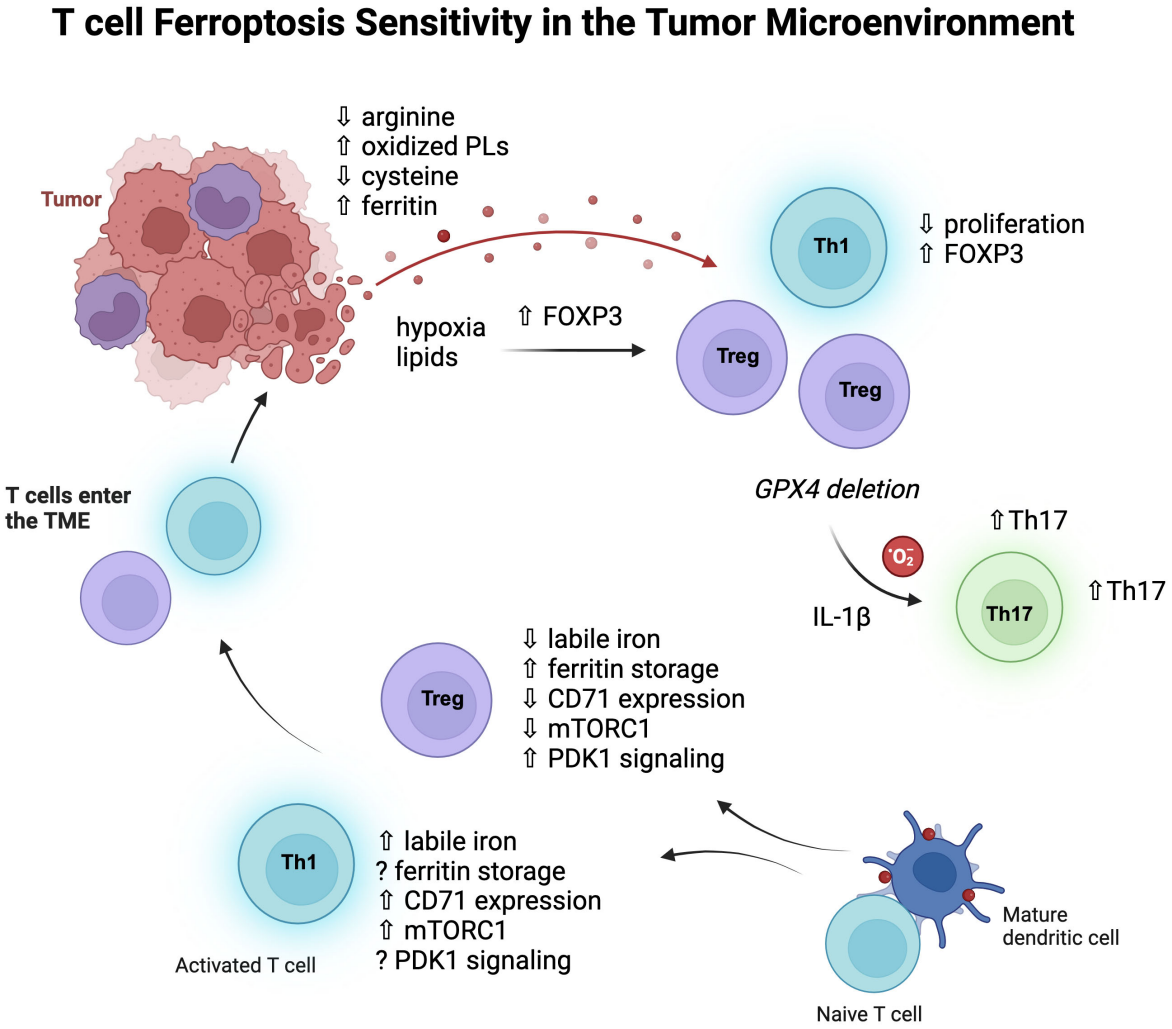
T cell subsets and ferroptosis sensitivity. Pro-inflammatory CD4 T cells, especially Th1 cells, contain more labile iron than Treg cells, express more transferrin receptor (CD71), and signal more through mTORC1. Iron homeostasis of Treg cells depends on the kinase PDK1 and upregulation of ferritin protein to store cellular iron and thereby lower labile iron. Once these cells enter the TME, the metabolic pressures of the environment can influence their metabolic programs and function. Hypoxia and lipid uptake promotes FOXP3 expression, and non-Treg cells can also express FOXP3, increasing Treg-like cells which have some survival advantages in the TME as they are less dependent on mTORC1 signaling and glycolysis. Tregs rely less on GPX4 than CD8 T cells at baseline but do depend on GPX4 expression for survival in tumors, where GPX4 deficiency rewires them to produce IL-1β and mitochondrial superoxide which then promotes Th17 cells.

Of all the CD4 subsets that have been investigated, regulatory T cells (Treg) appear to have the most distinct iron handling and metabolism (**Figure 2**). We have found that they contain less labile iron compared to pro-inflammatory Th1 cells, despite less CD71 expression (80). Furthermore, antibody-mediated blockade of induced Treg (iTreg) cultures did not reduce differentiation or viability and instead caused an increase in Foxp3 expression, their master regulatory transcription factor. Consistent with low levels of free iron, Wu et al. showed that Tregs express higher levels of ferritin heavy chain (FTH) than conventional T cells, which was required for the maintenance of a stable intracellular iron homeostasis (86). High FTH expression also influenced FOXP3 transcription by altering ten-eleven translocation (TET) dioxygenase demethylation activity of the FOXP3 locus. Therefore, disruption of this regulation could have the potential to destabilize Tregs in the TME.

Iron promotes glycolytic activity in T cells (87), and mTORC1 signaling (77), which generally Tregs rely less on for their metabolism (88, 89). Therefore, it is possible that lowering iron has less of an effect on Tregs due to their decreased dependence on glycolysis. However, recent work by Feng et al. showed that iron homeostasis in Tregs was regulated by MEK-ERK signaling which regulated CD71 expression, and this was independent of mTORC1 signaling (90). Here, deletion of the serine/threonine kinase 3-phosphoinositide-dependent protein kinase 1 (PDK1) in Tregs caused iron overload and excessive ROS, resulting in a fatal phenotype in mice. Interestingly, PDK1 knockout Tregs had increased rates of cell death, which was rescued by an iron chelator, although it is not certain whether this death was due to ferroptosis.

Genetic strategies of GPX4 deletion have significantly contributed to our understanding of ferroptosis in T cells. Interestingly, biallelic deletion of GPX4 using a CD4 promoter-Cre system significantly decreased CD8 T cell numbers in the periphery while keeping CD4 T cells intact (91). There was also no effect on the frequency of Tregs, providing evidence that different T cell subsets are differentially sensitive to ferroptosis *in vivo.* In another investigation using the CD4 promoter-Cre system, a vulnerability of Tfh cells for ferroptosis was uncovered during vaccination models (92). Therefore, inflammatory challenges and contexts have a major influence on relative T cell subset sensitivities to ferroptosis. In another example of this, GPX4 deletion in Tregs revealed a requirement for GPX4 in their survival upon stimulation (93). Although non-affected at steady-state, GPX4-deficient Tregs showed significantly elevated lipid peroxides and ferroptosis after activation. Interestingly, these Tregs also upregulated IL-1β, which stimulated Th17 responses. In a B16 melanoma model and MC38 colon carcinoma model, intratumoral GPX4-deficient Tregs also experienced ferroptosis, which lowered tumor burdens (93).

### 2.4 Natural killer cells

Natural Killer (NK) cells are innate lymphoid cells that possess highly cytotoxic and proinflammatory properties. Traditionally considered part of the innate immunity arm, NK cells also have capabilities associated with adaptive immunity, such as antigen-specific expansion and memory. In the TME, NK cells play a crucial role through both direct killing of tumors and the release of pro-inflammatory cytokines that further stimulate anti-tumor immune responses (94). Like other immune cells, NK cells in the TME are subject to immunosuppression and often become dysfunctional (95).

Iron is essential for the activation of NK cells. During acute infection, NK cells become highly glycolytic in an mTOR-dependent manner (96). Due to this increase in energy demand, NK cells also become dependent on iron, increasing CD71 expression. Inhibition of iron uptake via hepcidin treatment decreased the cytotoxicity of NK cells, highlighting the iron dependency of NK cell activation (97). However, disruptions in iron homeostasis such as during infection or cancer can also destroy NK cell function. In a model of gastric cancer, cancer-associated fibroblasts triggered ferroptosis in NK cells by increasing iron export in the TME and increasing the LIP, leading to iron overload and ferroptosis (98). Thus, iron concentrations in NK cells must be carefully regulated along a continuum of import and export like in other lymphocyte cell populations.

Lipid metabolism also mediates functional changes in NK cells that can impact anti-tumor immunity. In general, high lipid metabolism in NK cells lowers their function. Palmitic acid treatment of human NK cells showed decreased ROS, proliferation, IFN-γ and cytotoxicity (99). Furthermore, NK cells derived from B-cell lymphoma patients adapt to elevated serum lipids by increasing lipid metabolism show similar dysfunction, in part due to decreased glycolysis (100). Similarly, NK cells in obese patients upregulate the PPAR pathway, leading to lipid accumulation and worse anti-tumor responses (101). Given these data, it is plausible that an iron-starved, lipid-rich TME skews NK cells towards a dysfunctional state that blunts the anti-tumor immune response. Indeed, recent studies reveal that ferroptosis may play a role in NK cell dysfunction in the TME. NK cells co-cultured with ovarian cancer patient ascites become dysfunctional and have increased expression of lipid peroxidation and ferroptosis genes. However, stimulation of the Nrf2 antioxidation pathway rescued their function (102). This accumulating evidence shows that iron and lipid metabolism is central for NK cell function and calls for future studies on ferroptosis in NK cell biology.

### 2.5 B cells

B lymphocyte cells mediate adaptive immune responses through antigen presentation and antibody production. In autoimmune diseases such as systemic lupus erythematosus (SLE), B cells are generally considered pathogenic due to the production of autoantibodies. Interestingly, patients with SLE exhibit decreased B cell frequencies in peripheral blood, and B cell death in this setting has been shown to involve ferroptosis (103). Peripheral B cells cultured in SLE patient serum exhibited increased survival in the presence of ferroptosis inhibitors, whereas treatment of lupus mouse models with ferroptosis inhibitors decreased autoantibody-producing plasma cells (103–105). Therefore, ferroptosis may be involved in the differentiation of B cell subsets including antibody-secreting plasma cells. Indeed, B cell subsets have differential sensitivities to ferroptosis, with GPX4 being required for B1 and marginal zone B cell survival but extraneous in the development of B2 cells (106). Although ferroptosis inhibitors may have decreased plasma cells in the setting of SLE, SLE is also a disease with well-documented abnormalities in systemic and cellular iron metabolism, which may not translate to cancer patients (80, 107–109).

Despite multiple reports of B cells in the TME, the role of B cells in cancer is generally ill-defined, and sometimes controversial (110). Therefore, interpretation of ferroptosis sensitivities in different B cell subsets in the TME is difficult. Plasma cells, for example, show great heterogeneity revealed by single-cell RNA sequencing in cancer patients. In bladder cancer patient samples, plasma cell infiltration in the TME was associated with better overall survival and response to immunotherapy (111). Interestingly, single-cell RNA sequencing of head and neck cancer patient tumors also revealed multiple B cell populations including activated B cells, germinal center B cells, and plasma cells (112). Excitingly, the antigen specificity of some of these antibody-secreting B cells was shown to be human papillomavirus (HPV), which suggests that these antigen-specific B cells could be leveraged therapeutically within the TME of patients with HPV-positive head and neck cancer. Therefore, it is tempting to speculate based on the previous findings in autoimmune studies that in some cancer types ferroptosis inducing agents would not interfere with plasma cell differentiation or survival. Alternatively, pancreatic ductal adenocarcinoma (PDAC), which also demonstrates a TME rich with B cells, can harbor regulatory B cells (Bregs) that promote tumor growth and metastasis (113) and ferroptosis vulnerabilities in Bregs versus other B cell types requires further study.

### 2.6 Dendritic cells

Dendritic cells (DCs) are sentinel and professional antigen-presenting cells that migrate to tissues to sample and process soluble and cell-associated antigens. DCs assess their tissue environment for molecules secreted by stressed cells, microbial products, and metabolites (114). DCs are highly heterogeneous and are represented by multiple populations with differential developmental trajectories and immune functions. Simplified classification of DCs includes conventional type 1 DCs (cDC1) that can cross-present cell-associated antigens to CD8 T cells via Major Histocompatibility Complex class I (MHCI) after phagocytosing of infected or malignant cells, and conventional type 2 DCs (cDC2) that percent peptides from soluble antigens loaded at MHCII to prime CD4 T cells (115). cDC1 and cDC2 subsets activate anti-tumor immune responses by presenting tumor-associated antigens to CD4 and CD8 T cells (116).

Studies on ferroptosis in DCs are limited and based on models that need to account for the developmental and functional heterogeneity of DCs. GPX4 deficiency does not change the abundance of the total DC population in the spleen in mice (57). However, it remains unclear if GPX4 regulates DC subsets in other tissues or their capacity to migrate into draining lymph nodes and present antigens. Several lines of evidence suggest that lipid oxidation and ferroptosis might be a critical regulator of DC functions. First, in DCs, ROS produced by the NADPH-oxidase complex NOX2 drive lipid peroxidation and membrane damage in endosomes, which in turn results in antigen leakage into the cytosol and efficient antigen cross-presentation to CD8 T cells (117). This phenotype is abrogated when ROS is scavenged by α-tocopherol, but the effect of iron chelators was not tested in this study.

Second, CLEC9A, a receptor that binds dead-cell debris, has been shown to upregulate NOX2 activity and ROS-mediated phagosomal membrane damage in cDC1, also profoundly enhancing antigen cross-presentation to CD8 T cells (118). Interaction of DCs with CD4 T cells licenses so-called post-synaptic DCs to present antigens and efficiently generate robust CD8 T cell responses. Intriguingly, this process of licensing depends upon the induction of enhanced lipid peroxidation in DCs and inhibition of lipid peroxidation by α-tocopherol decreased activation of antigen-specific CD8 T cells (115). Although whether the lipid peroxidation processes are regulated by iron has not been tested in these three studies, it is possible that ferroptosis can regulate DC activation and licensing in CD8 T cell-mediated anti-tumor immunity.

In contrast to DC-intrinsic lipid peroxidation, cell-extrinsic lipid overload and oxidation in the TME can suppress the functions of DCs in mice. DCs that engulf ferroptotic, but not necroptotic, cancer cells show decreased inflammatory response and antigen presentation and fail to protect against tumor growth (119). Although it remains unclear how engulfing ferroptotic cells reprograms DC function, the cross-talk between tumor cells undergoing ferroptosis and DCs might alter tumor immunosurveillance. Tumor-derived oxidized lipids can decrease antigen presentation by DCs in mice by reducing MHCI expression (120). Interestingly, tumor-derived exosomes carrying excessive fatty acids to DCs contribute to decreased antigen presentation through the Peroxisome Proliferator Activated Receptor α (PPARα)-mediated metabolic reprogramming linked to enhanced lipid droplet biogenesis and fatty acid oxidation (121). Similarly, tumor-derived factors increase lipid peroxidation in mouse DCs to trigger the unfolded protein response (UPR) transcription factor XBP1 that increases lipid droplet biogenesis and suppresses antigen presentation (122). Finally, excessive fatty acids associated with obesity and a high-fat diet also increase mitochondrial ROS and the UPR in mouse DCs, resulting in altered DC function linked to the XBP1-dependent hyperinflammatory immune responses (123). Future studies are needed to decipher how different sources and mechanisms of lipid peroxidation in DCs depend upon iron and counterbalance DC functions in the TME.

## 3 Ferroptosis in the tumor microenvironment

**Figure 3:**
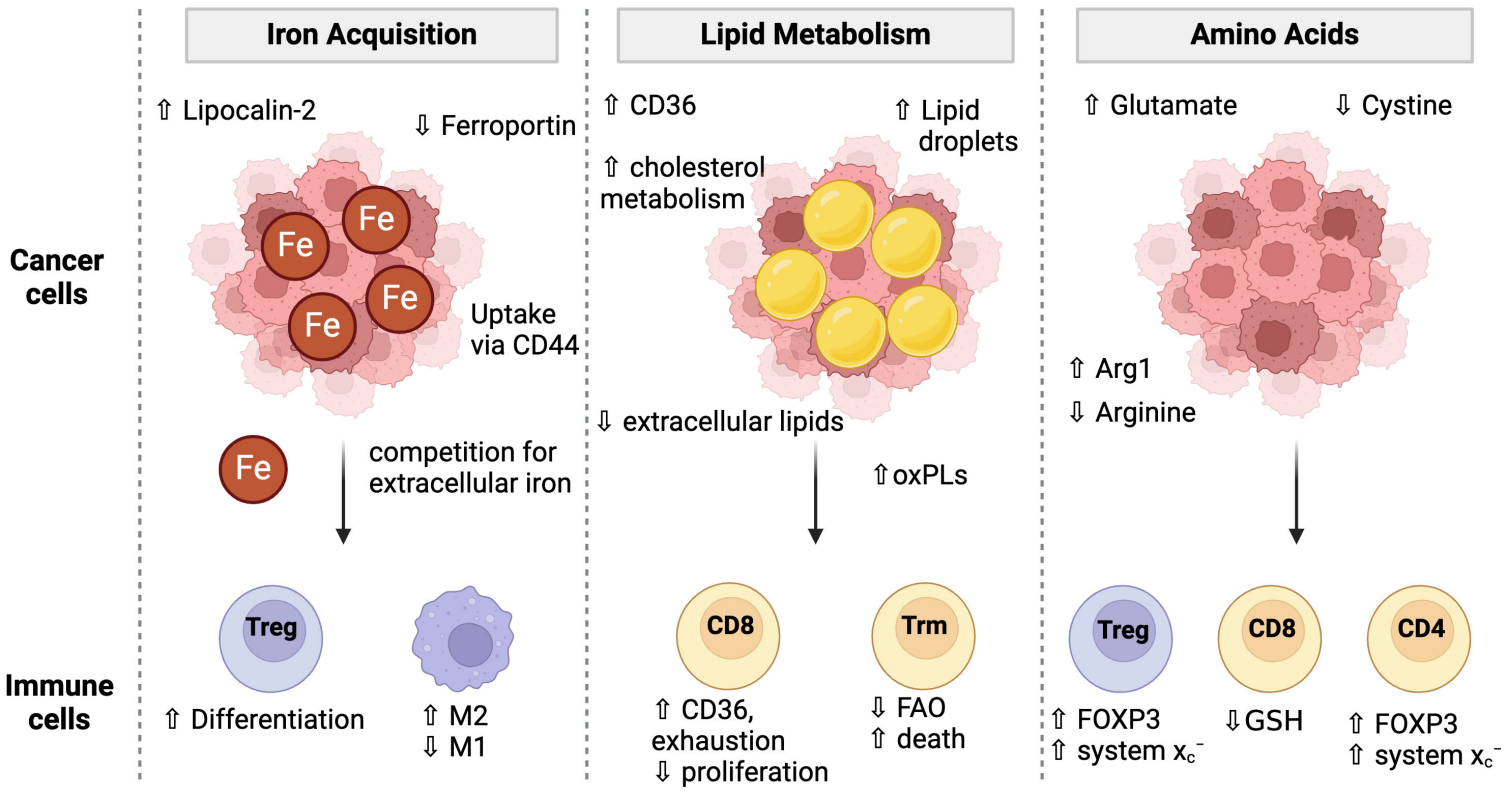
Cancer cell metabolism and conditioning of the TME for immune cells. Cancer cells adopt multiple strategies to fuel their proliferation and survival including the acquisition of iron, lipids, and amino acids. These metabolic programs condition the TME to have low iron and amino acids which then impacts immune cell metabolism and ferroptosis sensitivity. Cancer cells have unique iron uptake mechanisms such as lipocalin-2 and CD44 mediated uptake while simultaneously downregulating the iron exporter, Ferroportin. Low iron conditions favor Treg differentiation over Th1 or Th17 cells and promotes M1 over M2 macrophage differentiation. Cancer cells increase lipid uptake via CD36 and lipid droplet storage. The extracellular lipid environment is altered to contain a higher proportion of oxidized phospholipids which can cause toxicity to T cells and promote CD8 T cell exhaustion. Cancer cell metabolism high in Arginase-1 (Arg1) may deplete extracellular arginine. Low arginine promotes FOXP3 expression in CD4 T cells giving them suppressive activity. Low arginine and/or cystine causes T cells to upregulate the system X_c_^-^ to increase their GSH pools. However, cancer cell mediated cystine starvation ultimately leads to low GSH levels in T cells.

Although TME landscapes are extremely heterogeneous in terms of immune infiltration (defined as “hot” or “cold”), acidity, and oxygen content, there is increasing evidence that multiple cancer types develop unique resistance mechanisms to ferroptosis. In some cases, these adaptations also cause further conditioning of the TME that has consequences for the immune cell populations. Indeed, various pathways involved in ferroptosis become dysregulated in the tumor microenvironment (TME), namely those concerning lipid, amino acid, and iron metabolism (124, 125) (**Figure 3**). Hypoxic conditions, common in almost all solid tumors, promote ferroptosis resistance (88). Under these conditions, HIF-1α upregulates lactate dehydrogenase (LDH) and amino acid transporter SLC1A1. LDH increases lactate production, which confers resistance to ferroptosis in a pH-dependent manner. Simultaneously, the upregulation of SLC1A1 enhances glutamate uptake, which in turn boosts the efficiency of cystine uptake via the system x_c_^−^.

Competition for amino acids in the TME between immune cells and cancer cells can also influence ferroptosis sensitivity. Low arginine in the TME is common in many cancers and is associated with poor prognosis, in part due to poor T cell function (126). Low arginine in the TME has been shown to induce FOXP3 expression in CD4 T cells, which confers immunosuppressive function and promotes Tregs. However, Tregs become uniquely resistant to ferroptosis as they upregulate system x_c_^−^ expression to counteract the increased ROS from high mitochondrial respiration (74). In another example, arginine starvation in the TME impacts GSH levels and ROS signaling, which then influenced Treg function (127). However, arginine has been shown to augment erastin-induced ferroptosis (128) and modulating its uptake is useful in the TME where cancer cells adopt mechanisms to efficiently take up arginine (129), thus starving immune cells from arginine and potentially sparing them from ferroptosis.

Some TMEs which are acidic change the lipid uptake of cancer cells. An acidic pH promotes CD36 expression, lipid uptake, and lipid droplet formation across cancer cells from varying tissues (130). Because these acidic-adapted cancer cells rely more on lipid uptake in acidic conditions, this could be leveraged to induce ferroptosis. Excess fatty acids are typically stored within lipid droplets, but Dierge and colleagues showed that overwhelming this storage and buffering capacity with PUFAs can promote ferroptosis (131). In acidic colorectal cancer cells, omega-3 and omega-6 PUFAs selectively induced ferroptosis. Suppression of lipid droplet formation via diacylglycerol acyltransferase (DGAT) inhibition reduced lipid droplet biogenesis, leaving more PUFAs free from sequestration and promoting increased ferroptosis sensitivity. In another example of PUFA supplementation was applied to a model of intestinal-type gastric cancer (132). These cancer cells had lowered PUFA-synthesizing enzymes such as ELOVL5 and upregulated Ferroportin, conferring ferroptosis resistance. Supplementation with arachidonic acid restored ferroptosis sensitivity in these cells. Together, these models suggest a role for modulating lipid uptake in acidic TMEs to leverage ferroptosis induction in tumors.

Other cancers such as gastric cancer develop ferroptosis resistance through a unique mechanism involving cancer-associated fibroblast (CAF)-secreted exosomes (133). In this model, secreted exosomes containing miR-522 were taken up by gastric cancer cells. This downregulated ALOX15, a producer of lipid ROS in gastric cancer cells, and conferred resistance to chemotherapy. It is not clear whether these exosomes could also be taken up by local immune cells. However, we expect to see more examples emerge of secreted factors such as exosomes that influence ferroptosis sensitivity in both immune cells and cancer cells of the TME.

### 3.1 Lipid metabolism

Cancer cells can activate transcriptional programs to drastically modify the acquisition, storage, and handling of lipids to accommodate increased energy demands. Namely, cancers can upregulate CD36 to increase fatty acid (FA) uptake, increase FA synthesis, upregulate fatty acid oxidation (FAO) pathways, and increase the formation of lipid droplets to protect themselves from lipotoxic stress (134). The dysregulated lipid environment in the TME causes CD8 T cells to upregulate the CD36 lipid scavenger receptor, resulting in increased lipid uptake, lipid peroxidation, and the development of an exhausted phenotype (135, 136). FAO is required for some T cell subsets to adapt to the TME, including tissue-resident memory T cells (Trms). Trms co-cultured with gastric cancer cells were outcompeted and starved of fatty acids, resulting in their death (137). These data highlight the complexity of lipid metabolism in the TME, as lipids can be either beneficial or detrimental to immune cell-mediating killing of cancer cells.

Recent discoveries have focused on dysregulated lipid metabolism and the role it plays during ferroptosis and the metastatic process. Specifically, dysregulated cholesterol homeostasis has been implicated in resistance to ferroptosis, increasing tumorigenesis and metastasis. Chronic exposure of cells to 27-hydroxycholesterol, an abundant circulating cholesterol metabolite, selects for cells that exhibit increased cellular uptake or lipid biosynthesis and increased metastatic capability via stabilization of GPX4 (138). Another cholesterol metabolite, 7-Dehydrocholesterol (7-DHC), has also been implicated in ferroptosis sensitivity. 7-DHC is an intermediate metabolite of distal cholesterol biosynthesis that suppresses ferroptosis by shielding membrane lipids from autooxidation (139). Manipulating the biosynthesis pathway of 7-DHC or inhibiting the consumption of 7-DHC in cholesterol synthesis increased or decreased ferroptosis sensitivity, respectively. In a mouse xenograft model, blocking biosynthesis of 7-DHC lowered 7-DHC levels in the tumor tissues and reduced tumor growth (139). Together, these data suggest that cholesterol homeostasis may impact cancer pathogenesis by selecting for resistant cells or cholesterol intermediates may protect from ferroptosis, highlighting the importance of cholesterol metabolism in progression and metastasis.

Cancer cells may also be able to selectively incorporate certain lipid species into cellular lipid pools to modulate cell growth and metastatic potential. Henry and colleagues show that pB3 breast cancer cells rely on ether lipids incorporated into the cellular membrane to induce cancer stemness and metastasize (140). This allows breast cancer cells to uptake iron via CD44 in non-clathrin coated pits; a unique mode of iron acquisition. Increased intracellular iron allowed the cells to metastasize at the cost of being more susceptible to ferroptosis. These findings emphasize the potential connection between lipid and iron metabolism in the TME, allowing for growth but also rendering cells sensitive to ferroptosis.

### 3.2 Iron metabolism

All cells require some amount of iron for functions such as DNA replication and mitochondrial respiration. Cancer cells become dependent on iron uptake, as is required for various growth pathways. Hypoxia in the TME biases cancer cells toward an iron-scavenging state in which influx is upregulated and efflux is downregulated, effectively starving the TME of iron (141). Single-cell RNA-sequencing analysis of cerebrospinal fluid in patients with leptomeningeal metastases reveals that macrophages in the TME induce lipocalin-2 (LCN2) expression in cancer cells, which allows for enhanced iron uptake (142). LCN2 has been shown to play a role in pancreatic adenocarcinoma as well, stimulating the release of pro-inflammatory cytokines in the TME by binding to its receptor, SLC22A17 (143). Considering that LCN2 depletion increased survival in mouse models of tumors, its role in promoting inflammation can be considered deleterious, recruiting immune cells that quickly become dysfunctional.

As opposed to iron uptake, some cancer cells instead downregulate Ferroportin expression to retain more of their iron stores. Breast cancer cells downregulate Ferroportin allowing them to hold onto more iron (62). The iron-depleted state of the TME paired with increased intracellular iron of cancer cells makes targeting ferroptosis a potential strategy to uniquely target tumors. In fact, work by Alvarez and colleagues demonstrates that various cancer cell lines become dependent on NFS1-mediated generation of iron-sulfur clusters to meet increased metabolic and respiratory demands. Suppression of NFS1 in murine breast cancer xenografts augments the iron starvation response, increasing intracellular iron stores and sensitizing the cancer to oxidative damage and ferroptosis (144). Furthermore, ferroptosis induction can be leveraged in the treatment of neuroblastoma, a cancer characterized by increased intracellular iron. In this study, Withaferin A induced ferroptosis through the dual inhibition of GPX4 and activation of HO-1, increasing intracellular iron stores and causing lipid peroxidation and cell death (145).

## 4 The relationship between metastasis and ferroptosis

**Figure 4:**
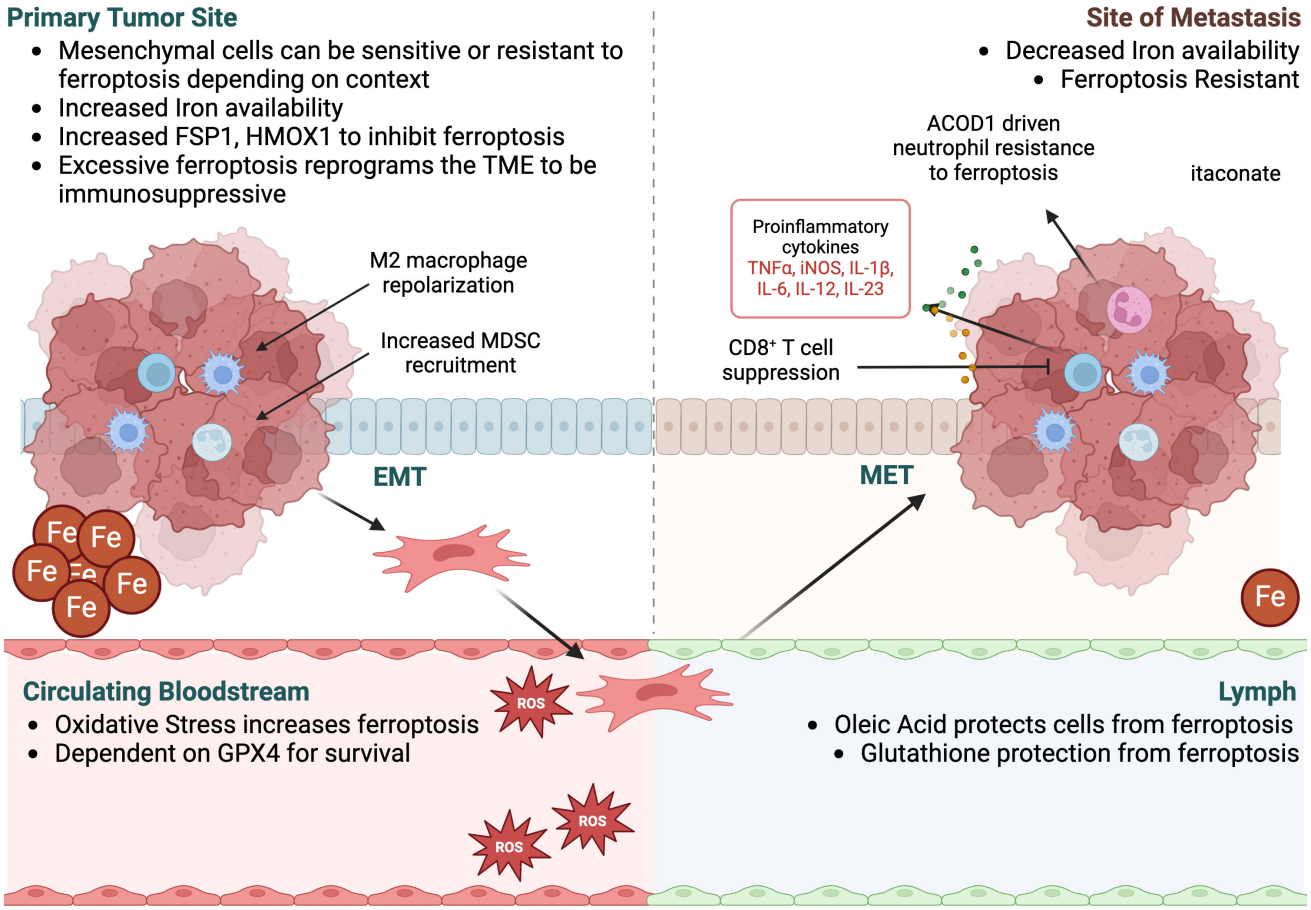
Ferroptosis in EMT and metastasis. Metastasis is characterized by the ability of tumor cells to undergo epithelial-to-mesenchymal transition (EMT), escape the primary tumor via the circulating bloodstream or lymph, and then enter the site of metastasis, where cells often undergo mesenchymal-to-epithelial transition (MET) to form secondary distant tumors. Ferroptosis plays a role in each stage of metastasis, both within the cancer cells and the surrounding TME. While ferroptosis sensitivity of the tumor cells at each stage is dependent on tissue context, excessive ferroptosis within the primary tumor can reprogram the TME to be immunosuppressive, supporting tumor growth and metastasis. Once in the bloodstream, oxidative stress increases ferroptosis of the tumor cells, as they become more dependent on GPX4 for survival. Alternatively, cells that travel to sites of metastasis through lymph are protected from ferroptosis due to an environment rich in oleic acid and glutathione, reducing oxidative stress. At distant sites of metastases, iron availability is typically lower than the primary tumor and tumor cells are more resistant to ferroptosis. Itaconate levels are increased by the presence of neutrophils and potentially macrophages at these sites due to their high expression of ACOD1, supporting immune suppression and tumor growth within the metastatic site.

The relationship between metastasis and ferroptosis is another active area of study. Metastasis is a major cause of cancer-related mortality as cancer cells spread throughout the body, making treatment more challenging. Within this landscape, cancer cells, including those involved in metastasis often exhibit alterations in iron metabolism and lipid peroxidation pathways, which can make them or less susceptible to ferroptosis. Resistance to this mode of cell death not only fosters heightened cell survival but also metastatic expansion and resistance against therapy. Ferroptosis can influence multiple aspects of metastasis through processes such as epithelial-to-mesenchymal (EMT) transition, metabolic rewiring, and tumor microenvironment remodeling for immune suppression (**Figure 4**). While the precise mechanisms linking ferroptosis to metastasis are still an active area of research, current evidence suggests that ferroptosis plays a role in cancer progression and metastasis, thus positioning it as a promising therapeutic avenue for combatting metastatic disease.

### 4.1 Epithelial-to-mesenchymal transition

EMT is characterized by epithelial cells losing cell-to-cell adhesion to acquire migratory and invasive capabilities, facilitating infiltration into the surrounding stroma, escape from the primary tumor, intravasation into the bloodstream, and subsequent extravasation at distant sites, resulting in the establishment of macrometastases (146). This phenomenon, underscored by its plasticity, serves as a survival mechanism for tumor cells in adverse environmental conditions, including chemotherapy and metabolic stress (147). As such, ferroptosis and the resistance thereof, has been linked to the regulation of EMT markers and pathways. Notably, Viswanathan and colleagues showed that high-mesenchymal state melanomas relied on ferroptosis resistance *in vivo*, a trend replicated across various *in vitro* models of high-mesenchymal state human cancers, underscoring EMT-driven ferroptosis resistance as a key survival mechanism (148). Furthermore, investigations by Kauffman and colleagues revealed a disparity in iron levels between primary renal cell carcinoma (RCC) tumors and metastases, where primary RCC tumors were shown to have higher iron content than other solid tumors, with significantly lower levels as the tumors become metastatic (149), suggesting increased resistance to ferroptosis during metastatic progression. Deficiency of AMER1, a regulator of the WNT signaling pathway that controls EMT, has also been shown to decrease the pool of labile free iron, thereby increasing ferroptosis resistance in colorectal cancer cells, and subsequent survival and metastasis (150).

On the other hand, there have also been multiple studies illustrating that EMT high cells have increased sensitivity to ferroptosis induction (151–154). Upregulation of Bach1, a transcription factor regulating heme oxygenase-1 (HO-1) which catalyzes the breakdown of heme into iron, correlated with EMT and ferroptosis sensitivity in glioma cells, suggesting Bach1 as a potential marker of ferroptosis susceptibility in glioma patients (152). Similarly, in EMT-high lung cancer cell lines, Wang et al., showed that the E3 ubiquitin ligase MIB1 promotes NRF2 degradation, increasing reactive oxygen species production and rendering cells more susceptible to ferroptosis inducers (153). Additionally, the downregulation of E-Cadherin, a hallmark of mesenchymal cells, enhanced ferroptosis sensitivity, further underscoring the intricate interplay between EMT and ferroptosis regulation (154).

These seemingly conflicting data highlight the complexity of both EMT and ferroptosis. It’s possible that the genetic context in which EMT occurs may account for differential sensitivity to ferroptosis. For example, p53, a cell-cycle arrest protein commonly lost in metastatic cancers including colon and pancreatic cancers, inhibits cysteine uptake and sensitizes cells to ferroptosis, suggesting cancer cells lacking p53 may be more resistant to ferroptosis (155). Additionally, KRAS-mutated cells have been shown to upregulate FSP1 to resist ferroptosis-inducers in non-small cell lung cancer, colorectal cancer, and pancreatic cancer which have elevated levels of FSP1 (156). Interestingly, FSP1 also increased 3D spheroid growth in KRAS WT cells, suggesting ferroptosis resistance alone could promote tumorigenesis and metastasis.

### 4.2 GPX4

GPX4 can influence metastasis through several mechanisms including the reduction of oxidative stress, inhibition of ferroptosis regulation of signaling pathways, and interactions within the tumor microenvironment. GPX4-mediated inhibition of ferroptosis may suppress metastatic dissemination by protecting cancer cells from ferroptotic cell death and as such, several proteins have been shown to protect cancer cells from undergoing ferroptosis to promote tumorigenesis and metastasis, through the regulation of GPX4. CST1, a cysteine protease inhibitor, stabilizes GPX4, to promote gastric cancer metastasis (157). SGK2, a serum glucocorticoid regulated kinase, was also shown to upregulate GPX4 expression in prostate cancer, promoting survival and metastasis (158). GPX4 can also modulate signaling pathways associated with metastasis, including PI3K/AKT, MAPK/ERK, and NF-κB pathways, through its antioxidant activity and interactions with redox-sensitive signaling molecules (159). Dysregulation of these pathways can promote cancer cell survival, proliferation, and metastasis.

Once the cancer cells have escaped the primary tumor and entered the blood stream, cells undergo oxidative stress and experience high levels of cell death (160), likely including ferroptosis. Cancer cells in the blood stream that have enhanced redox capacity and therefore a metabolic advantage are more likely to resist cell death, including ferroptosis, and form macrometastases at the metastatic site. For example, dietary antioxidants have been shown to reduce oxidative stress and increase metastasis in models of lung cancer and melanoma (161, 162). These data suggest that the metastatic route may also play a role in ferroptotic cell death resistance. As such, Ubellacker et al. showed that melanoma cells in the lymph experience less oxidative stress and form more metastases than melanoma that metastasize via the blood stream which were dependent on GPX4 (163). The authors showed that lymph fluid had lower levels of glutathione and oleic acid, which protected melanoma cells from ferroptosis, supporting additional research investigating the protection of cells from ferroptosis via oleic acid (164).

GPX4 may modulate EMT by regulating oxidative stress and redox signaling pathways involved in EMT induction and maintenance. Oxidative stress can promote metastasis by inducing DNA damage, genomic instability, and the activation of signaling pathways associated with cancer cell migration, invasion, and metastatic spread. Reactive oxygen species (ROS) is essential of cancer cell survival, however, excess ROS can cause oxidative stress and lead to cancer cell death (165). Additionally, the production of mitochondrial ROS within the cancer cells has been suggested to drive (166, 167) or reduce (168, 169) EMT and the metastatic potential of cancer cells, again highlighting the complexity of metastasis and the likelihood that this process is dependent on the context and local tumor microenvironment (48).

### 4.3 Tumor microenvironment reprogramming

Ferroptosis can affect the composition and function of the tumor microenvironment, including immune cells, stromal cells, and extracellular matrix components to influence metastatic potential through several mechanisms including immunosuppression, macrophage polarization, and cytokine signaling. Ferroptosis-induced cell death leads to the release of damage-associated molecular patterns (DAMPs) and pro-inflammatory signals, triggering an immune response (170, 171). However, sustained or excessive ferroptosis may result in immunosuppression by depleting immune cells or inducing dysfunction in immune populations, such as T cells, natural killer (NK) cells, and dendritic cells. Immunodeficiency can impair the ability of the immune system to detect and eliminate cancer cells, thereby facilitating metastasis. For example, Drijvers et al. showed that activated T cells were significantly more sensitive to RSL3-induced ferroptosis than cancer cells (172). Additionally, ferroptosis can regulate the production and secretion of cytokines and chemokines involved in immune cell recruitment, activation, and polarization and therefore dysregulated ferroptosis in cancer cells may alter the cytokine and chemokine profile of the tumor microenvironment, promoting the recruitment of pro-tumorigenic immune cell populations and creating a metastasis-permissive niche.

Dysregulation of ferroptosis in tumor cells may influence the presentation of tumor antigens and the activation of cytotoxic T lymphocytes (CTLs), which are critical for tumor surveillance and metastasis prevention. Additionally, ferroptosis-induced immunogenic cell death may enhance the efficacy of immunotherapy by stimulating antitumor immune responses. Another immune cell type influenced by ferroptosis are MDSCs, which have been shown to promote tumor progression, metastasis, and immunotherapy resistance (173). Ferroptosis-induced tissue damage and inflammation can recruit MDSCs to the tumor site and enhance their immunosuppressive activity, impairing antitumor immune responses and facilitating metastatic dissemination. For example, ferroptosis can increase the expression of HMGB1, a non-histone nuclear protein that regulates immune response by binding to immune receptors such as RAFE and TLR9 (174, 175), which in turn increases the expression of CXCL10 on cancer cells to recruit CD8 T cells as well as immunosuppressive MDSCs, but also upregulate PD-L1. PD-L1 immune checkpoint inhibitors blocking the MDSC-mediated suppression of CD8 T cells reduced liver metastases in colorectal cancer mouse models (176), highlighting the role of ferroptosis-recruited MDSCs in metastasis.

Ferroptosis can influence the polarization of TAMs, a key component of the tumor immune microenvironment. Depending on the context, ferroptosis may promote the polarization of TAMs toward a pro-tumorigenic phenotype (such as M2-like macrophages) that supports tumor growth, angiogenesis, and metastasis. For example, annexin A3 (ANXA3)-positive exosomes are secreted from TAMs, which can reprogram macrophages to an M2-like phenotype and inhibit ferroptosis of laryngeal squamous cell carcinoma cancer cells to promote metastasis to the lung (177). Alternatively, ferroptosis-induced inflammation and tissue damage may activate TAMs to produce pro-inflammatory cytokines and chemokines that recruit immune cells and promote metastasis.

Aconitate decarboxylase 1 (Acod1) catalyzes the reaction from cis-aconitate to itaconate and is commonly expressed in macrophages and neutrophils and induced by pathogens, toll-like receptor (TLR) ligands, as well as inflammatory cytokines (178, 179). Tumor-infiltrating neutrophils upregulate Acod1 expression to produce itaconate, which mediates Nrf2-dependent resistance to ferroptosis for neutrophil survival, which in turn can drive metastasis (180). Acod1 ablation in neutrophils resulted in ferroptosis and thus decreased metastases and increased anti-tumor immunity and immune checkpoint blockade efficacy. These results implicate neutrophil ferroptosis resistance as a mechanism of immune suppression in driving metastasis. Given that immunosuppressive macrophages can also express Acod1 to resist ferroptosis, is it likely Acod1 and its metabolic product itaconate might play a role in metastasis since immunosuppressive macrophages have been shown to drive metastasis. Further research is needed to decipher this relationship.

Overall, ferroptosis can influence immune cells in the tumor microenvironment through various mechanisms, potentially promoting metastasis by modulating immune responses, inflammation, and immunosuppression. Further research is needed to elucidate the specific roles of ferroptosis in immune cell function and metastatic progression and to explore its therapeutic implications for cancer treatment.

## 5 Immunomodulatory therapeutic opportunities in ferroptosis

Immune cell function, ferroptosis, and cancer immunotherapies are intertwined by metabolic demands and competition. Indeed, changing the TME through targeting metabolism is a popular strategy to improve cancer therapies (181). To this end, combining immunotherapies with ferroptosis-modulating small molecules is also being explored (182, 183). For example, immunotherapy using anti-tumor CD8 T cells showed in mice that CD8 T cells can enhance lipid peroxidation in the TME and contribute to ferroptosis in the tumor cells (184). Interferon (IFN)-γ released from activated T cells caused downregulation of the system x_c_^−^ expression in tumor cells, sensitizing them to ferroptosis. This highlights the potential for combining immunotherapies that enhance immune cell effector functions to simultaneously modulate ferroptosis sensitivity in other cells in the TME.

Another strategy for perturbing the metabolic landscape of the TME is manipulation of local iron availability with nanoparticles (185). The use of ferumoxytol, an iron oxide nanoparticle approved for the treatment of iron deficiency, was able to overwhelm Ferroportin-low AML tumors (186) and repolarize TAMs to an M1-like phenotype in murine adenocarcinoma models (187). Overall, these studies show that iron composition in the TME can be targeted to enhance ferroptosis induction in cancer and reprogram anti-tumorigenic immune cell responses.

Other favorable approaches to exploiting ferroptosis in cancer treatment involve strategies to induce high levels of ROS in the TME, further sensitizing cells to ferroptosis. These ROS-based therapies can include photodynamic therapy, sonodynamic therapy, and chemodynamic therapy. Increasing oxygen content in hypoxic TMEs is especially promising, although the precise amount needs to be carefully titrated to avoid activating survival-based stress response pathways in cancer cells (188). Phototherapy is one strategy in which nanoparticles are selectively incorporated into tumors and induce ROS intratumorally (189). Chlorin e6, a potent FDA-approved photosensitizer that is selectively taken up by metabolically-challenged tumors (190) has been combined with erastin, a system x_c_^−^ inhibitor, to generate a supramolecular nanodrug capable of inducing ferroptosis in cancer cells (191). Briefly, the nanodrug enhanced the efficacy of phototherapy in a model of squamous cell carcinoma by promoting ROS generation specifically in tumor cells. Chlorin e6 has also been combined with an Nrf2 inhibitor and ROS-generating MnO_2_ to inhibit the Nrf2-based defense pathway under oxidative stress and promote ferroptosis (192). Other ROS-inducing therapies such as sonodynamic therapy which uses ultrasound technology have been used to leverage ferroptosis sensitivity in cancer cells. For example, intratumoral injection of a platinum-cyanine small molecule complex in a murine 4T1 breast cancer model was able to trigger ferroptosis via depletion of GSH and GPX4 (193). Interestingly, sonodynamic therapy may also improve therapeutic responses in “cold” solid tumors, including checkpoint immunotherapies (194). In another phototherapy using Y8 nanoparticles, growth of both primary and metastatic tumors were reduced in mice and increased CD8 T cell responses (195). Thus, combining ferroptosis-inducing agents with approved phototherapy drugs or ROS-generating agents can overcome the limitations of some therapies in the treatment of hypoxic cancers.

### 5.1 Challenges

A major bottleneck in leveraging ferroptosis in cancer therapy comes from difficulties in “drugging” ferroptosis pathways (196). Popular strategies for inducing ferroptosis include erastin, and RSL3. However, erastin has limited solubility and is susceptible to metabolic degradation. To improve this, erastin analogs such as imidazole ketone erastin (IKE) have been developed. IKE had improved potency and stability in a xenograft model of diffuse large B cell lymphoma (197), although toxicity of free IKE was notable. Despite this, erastin has shown the ability to increase the susceptibility of cancer cells to chemotherapy drugs, at least *in vitro*.

RSL3 is well-tolerated in many mouse models of tumors and shows promise in intratumoral injection models but similarly suffers from poor solubility and pharmacokinetics, making it an unlikely therapy for the treatment of cancer. Derivatives of RSL3, therefore, have been extensively explored. However, both stereoisomers of RSL3, (S)-RSL3 and (R)-RSL3, show similar effectiveness in killing colorectal cancer (CRC) cells, despite (R)-RSL3’s inability to inhibit GPX4. This suggests that RSL3’s can induce cell death independently of GPX4 inhibition, acting instead as a broad inhibitor of the selenoproteome including thioredoxin peroxidase systems (198). Therefore, there remain significant challenges in our understanding of specificity and the mechanism of action of ferroptosis inducers, especially those targeting GPX4.

Inducing ferroptosis by non-pharmacological means is also possible but has uncovered resistance mechanisms. For example, ionizing radiation (IR) can induce ferroptosis in cancer cells by both increasing ROS levels and expression of ACSL4 (199). However, IR-treated tumors can develop resistance through upregulation of GPX4 and the system x_c_^−^. However, understanding these resistance mechanisms can allow for an additional layer of targeting with the respective inhibitors such as the earlier example of Nrf2 inhibitors and Chlorin e6 therapy in pancreatic cancer (192). Despite the success of ROS-inducing therapies such as these, this raises larger questions about how ROS generation will impact the immune cells in the TME. Most studies have focused on cancer cell death outcomes and therefore information is limited on the immune cell side. Ideally, generating high ROS would enhance cDC1 cross-presentation, increase M1 macrophage differentiation, and push Tregs into a state of excessive damaging ROS. Indeed, some near-infrared photoimmunotherapy (NIR-PIT) experiments have directed immune-specific effects based on the targeting monoclonal antibody to induce DC activation or deplete Tregs in the TME (200).

Another challenge for testing ferroptosis and uncovering potential mechanisms of resistance is that cell culture experiments do not translate *in vivo*, where the metabolic and/or immunosuppressive conditions of the TME can change or alter phenotypes. Extrinsic factors that can regulate ferroptosis resistance in cancer cells such as micronutrients, damage-associated molecular patterns (DAMPs), and metabolites are typically not recreated *in vitro* (201). Modifications to media formulations such as supplementation with selenium or depletion of arginine may offer some improvements. Another exciting possibility are patient-derived organoids that can retain some of their metabolic phenotypes *ex vivo* (202).

## 6 Discussion

To bias ferroptosis towards tumors and not immune cells achieving selective targeting is technically challenging. It remains chiefly unclear how to trigger ferroptosis in specific immune cell populations, creating a more favorable TME for efficient anti-tumor responses. To address these questions, an improved understanding of ferroptosis in different immune cell subsets is required. There are many unknowns remaining as some cell types have not yet been studied with respect to ferroptosis.

As far as some of the newer investigations into iron metabolism and ferroptosis, we expect to see many “new” or “non-canonical” ways to alter ferroptosis distinct from the canonical GPX4 inhibition. Along these lines, we expect to see how genes mutated or dysregulated in cancer cells can guide the discovery of novel mechanisms of ferroptosis. For example, mechanisms of AFG3L2 and SLC25A39 regulation was just elucidated over the past few years, and it would not be surprising to see these genes upregulated or mutated in cancers to bypass increased oxidative stress at the mitochondria.

If the metabolic demands for both cancer cells and immune cells in specific tissue niches are known, we could uncover other ways to condition the TME for ferroptosis that are tissue-specific, or specifically prevent metastases. With this knowledge, dietary modifications can be made to modulate those metabolite availabilities to preferentially favor anti-tumorigenic immune cell populations. For example, a ketogenic diet (high fat, low carbohydrate) in mice delayed tumor growth but accelerated cachexia (203). However, the diet increased lipid peroxidation and depleted GSH levels in cancer cells leading to ferroptosis. In another example of lipid metabolism, mice fed PUFA-rich diet compared to a MUFA-rich diet incorporated more PUFAs into xenograft cancer cells and responded better to ferroptosis-inducing agents (131). Dietary PUFAs from the fish-oil supplemented diet was sufficient to increase PUFA-lipids in the serum and promote an anti-cancer effect which was associated with lipid peroxidation in the tumor. Manipulation of dietary selenium was also shown to impact ferroptosis sensitivity in immune cells (92). Supplementation of selenium increased GPX4 expression in murine T cells, which promoted Tfh cell functions in a vaccination model by reducing their ferroptosis. Furthermore, selenium supplementation also increased GPX4 expression in human T cells and Tfh responses during influenza vaccination. Although not tested in a tumor model, this showed the potential of modulating immune cell ferroptosis sensitivity through diet.

Finally, ferroptosis cannot be defined as “good” or bad” but may be context-dependent and an important regulator of tissue homeostasis. Additionally, we cannot assume that a gene like GPX4 is the end-all-be-all of ferroptosis sensitivity. Despite its central role in ferroptosis, there is a lack of clear understanding how GPX4 regulates multiple processes in the cell, potentially varying greatly in different cancer types or immune cell populations. For example, as more attention is focused on GPX4 biology, additional roles of GPX4 have been uncovered for regulation of signaling pathways independent of ferroptosis, such as STING activation (204). Clearly, the ferroptosis-associated metabolic enzymes are not mediating one reaction but can influence various signaling pathways and execute potential “moonlighting” functions.
